# Supplementary material for: Cell cycle regulation of the psoriasis associated gene CCHCR1 by transcription factor E2F1
Source: PLoS One. 2023 Dec 21;18(12):e0294661. doi: 10.1371/journal.pone.0294661 (PMC10734992; doi:10.1371/journal.pone.0294661)
Supplement: S1 Table — (PDF) [file pone.0294661.s001.pdf]

Transcription factor	Matrix[A]	Detailed Matrix Information	Matrix similarity[B]	Strand	Position[C]
GABP	V$GABPB1.01	GA repeat binding protein, beta 1	0.807	(-)	-1208 to -1188
	V$GABP.01	GABP: GA binding protein	0.889	(+)	+79 to +99
MYC	V$CMYC.02	Myelocytomatosis oncogene (c-myc proto-oncogene)	0.940	(+)	-271 to -255
NFY	V$NFY.04	Nuclear factor Y (Y-box binding factor)	0.925	(+)	-913 to -899
	V$NFY.04	Nuclear factor Y (Y-box binding factor)	0.939	(+)	-710 to -696
	V$NFY.04	Nuclear factor Y (Y-box binding factor)	0.962	(-)	-257 to -243
	V$NFY.03	Nuclear factor Y (Y-box binding factor)	0.864	(-)	-184 to -170
YY1	V$YY1.02	Yin and Yang 1 repressor sites	0.944	(-)	+299 to +321

S1 Table. Putative transcription factor binding sites in the *CCHCR1-TCF19* bidirectional promoter. [A]: MatInspector library: Matrix Family Library Version 9.2 (January 2015). [B]: A score of 1.00 indicates a perfect match to the matrix. A “good” match to the matrix has a score > 0.80. [C]: Numbers refer to the position related to the transcription start site (+1) of *CCHCR1* (NCBI Reference Sequence: NM_019052.3).
