# Supplementary material for: Cell cycle regulation of the psoriasis associated gene CCHCR1 by transcription factor E2F1
Source: PLoS One. 2023 Dec 21;18(12):e0294661. doi: 10.1371/journal.pone.0294661 (PMC10734992; doi:10.1371/journal.pone.0294661)
Supplement: S2 Table — (PDF) [file pone.0294661.s002.pdf]

Matrix[A]	Detailed Matrix Information	Matrix similarity[B]	Strand	Position[C]
V$E2F.02	E2F, involved in cell cycle regulation, interacts with Rb p107 protein	0.844	(+)	-936 to -920
V$E2F4.01	E2F transcription factor 4, p107/p130-binding protein	0.978	(+)	-863 to -847
V$E2F4_DP2.01	E2F-4/DP-2 heterodimeric complex	0.799	(-)	-594 to -578
V$E2F.03	E2F, involved in cell cycle regulation, interacts with Rb p107 protein	0.853	(+)	-350 to -334
V$E2F2.01	E2F transcription factor 2	0.882	(-)	-340 to -324
V$E2F2.01	E2F transcription factor 2	0.888	(+)	-339 to -323
V$E2F3.02	E2F transcription factor 3 (secondary DNA binding preference)	0.892	(-)	-292 to -276
V$RB_E2F1_DP1.01	RB/E2F-1/DP-1 heterotrimeric complex	0.766	(+)	-289 to -273
V$E2F4.01	E2F transcription factor 4, p107/p130-binding protein	0.967	(-)	-279 to -263
V$E2F4_DP1.01	E2F-4/DP-1 heterodimeric complex	0.974	(-)	-233 to -217
V$E2F4_DP1.01	E2F-4/DP-1 heterodimeric complex	0.965	(+)	-232 to -216
V$E2F3.02	E2F transcription factor 3 (secondary DNA binding preference)	0.863	(+)	-230 to -214
V$E2F1_DP2.01	E2F-1/DP-2 heterodimeric complex	0.861	(-)	-205 to -189
V$E2F.01	E2F, involved in cell cycle regulation, interacts with Rb p107 protein	0.858	(-)	-168 to -152
V$E2F.03	E2F, involved in cell cycle regulation, interacts with Rb p107 protein	0.887	(+)	-133 to -117
V$E2F3.01	E2F transcription factor 3	0.903	(+)	-30 to -14
V$E2F3.01	E2F transcription factor 3	0.864	(-)	-29 to -13
V$E2F.03	E2F, involved in cell cycle regulation, interacts with Rb p107 protein	0.870	(+)	-13 to +4
V$E2F4.01	E2F transcription factor 4, p107/p130-binding protein	0.979	(+)	+6 to +22
V$E2F4.01	E2F transcription factor 4, p107/p130-binding protein	1.000	(+)	+99 to +115
V$E2F4.01	E2F transcription factor 4, p107/p130-binding protein	0.969	(+)	+480 to +496

S2 Table. Putative E2F binding motif in the *CCHCR1-TCF19* bidirectional promoter. [A]: MatInspector library: Matrix Family Library Version 9.2 (January 2015). [B]: A score of 1.00 indicates a perfect match to the matrix. A "good" match to the matrix has a score > 0.80. [C]: Numbers refer to the position related to the transcription start site (+1) of *CCHCR1* (NCBI Reference Sequence: NM_019052.3).
