# Supplementary material for: Cell cycle regulation of the psoriasis associated gene CCHCR1 by transcription factor E2F1
Source: PLoS One. 2023 Dec 21;18(12):e0294661. doi: 10.1371/journal.pone.0294661 (PMC10734992; doi:10.1371/journal.pone.0294661)
Supplement: S3 Table — (PDF) [file pone.0294661.s003.pdf]

Genevestigator microarray dataset: Perturbations				
**Rank**	**Gene**	**Description**	**Pearson's correlation coefficient**	**Up-regulated in G1/S or S phase?**
1	*CCHCR1*	coiled-coil alpha-helical rod protein 1	0.6324975	Yes (This study)
2	*CCHCR1*	coiled-coil alpha-helical rod protein 1	0.5560147	Yes (This study)
3	*CCHCR1*	coiled-coil alpha-helical rod protein 1	0.5327725	Yes (This study)
4	*RECQL4*	RecQ protein-like 4	0.44209898	Yes [1]
5	*NRM*	nurim (nuclear envelope membrane protein)	0.41889465	NYD
6	*KIFC1*	kinesin family member C1	0.40744227	NYD
7	*WRAP53*	WD repeat containing, antisense to TP53	0.39196926	NYD
8	*ASF1B*	ASF1 anti-silencing function 1 homolog B (S. cerevisiae)	0.39032698	Yes [2]
9	*RAD54L*	RAD54-like (S. cerevisiae)	0.3883079	NYD
10	*EME1*	essential meiotic endonuclease 1 homolog 1 (S. pombe)	0.38170868	NYD
11	*ZDHHC12*	zinc finger, DHHC-type containing 12	0.38015762	NYD
12	*PKMYT1*	protein kinase, membrane associated tyrosine/threonine 1	0.3774253	Yes [3]
13	*TCF19*	transcription factor 19	0.37672827	Yes [4] (This study)
14	*DDX11*	DEAD/H (Asp-Glu-Ala-Asp/His) box helicase 11	0.373824	NYD
15	*RECQL4*	RecQ protein-like 4	0.37250307	Yes [1]
16	*DDX11*	DEAD/H (Asp-Glu-Ala-Asp/His) box helicase 11	0.37225822	NYD
17	*STRA13*	stimulated by retinoic acid 13 homolog (mouse)	0.37041822	Yes [5]
18	*TROAP*	trophinin associated protein (tastin)	0.37041137	NYD
19	*C16orf59*	chromosome 16 open reading frame 59	0.3693077	NYD
20	*E2F1*	E2F transcription factor 1	0.36807078	Yes [6]
21	*RDM1*	RAD52 motif 1	0.3665757	NYD
22	*BRCA1*	breast cancer 1, early onset	0.36540088	Yes [7]
23	*MXD3*	MAX dimerization protein 3	0.36121294	Yes [8]
24	*TONSL*	tonsoku-like, DNA repair protein	0.3605641	NYD
25	*FAM203A*	family with sequence similarity 203, member A	0.35827172	NYD

Genevestigator microarray dataset: Cell lines			
**Rank**	**Gene**	**Description**	**Pearson's correlation coefficient**
1	*RNASEH2A*	ribonuclease H2 subunit A	0.50694025
2	*NELFE*	negative elongation factor complex member E	0.49831852
3	*DHX16*	DEAH-box helicase 16	0.49187893

4	*KIF2C*	kinesin family member 2C	0.4866221
5	*BAG6*	BCL2 associated athanogene 6	0.4837167
6	*TUBB*	tubulin beta class I	0.47268584
7	*CDC20*	cell division cycle 20	0.46996227
8	*CHAF1B*	chromatin assembly factor 1 subunit B	0.4682017
9	*CDC25C*	cell division cycle 25C	0.46737617
10	*TCF19*	transcription factor 19	0.4619712
11	*KIF4A*	kinesin family member 4A	0.46129447
12	*AURKB*	aurora kinase B	0.45510256
13	*PTTG1*	pituitary tumor-transforming 1	0.44862267
14	*SPAG5*	sperm associated antigen 5	0.44852474
15	*TUBBP2*	tubulin beta pseudogene 2	0.44831976
16	*FKBPL*	FK506 binding protein like	0.4475556
17	*NRM*	nurim (nuclear envelope membrane protein)	0.44644895
18	*CSNK2B, XXbac-BPG32J3.22*	CSNK2B:casein kinase 2 beta	0.44583622
19	*TPX2*	TPX2, microtubule-associated	0.441935
20	*DONSON*	downstream neighbor of SON	0.44076356
21	*DSN1*	DSN1 homolog, MIS12 kinetochore complex component	0.43877697
22	*PARP2*	poly(ADP-ribose) polymerase 2	0.438576
23	*ABCF1*	ATP binding cassette subfamily F member 1	0.43632245
24	*GTF2H4*	general transcription factor IIH subunit 4	0.43618095
25	*MEA1*	male-enhanced antigen 1	0.43477845
**Genevestigator microarray dataset: Cancers**			
**Rank**	**Gene**	**Description**	**Pearson's correlation coefficient**
1	*TSEN54*	tRNA splicing endonuclease subunit 54	0.55687207
2	*ASF1B*	anti-silencing function 1B histone chaperone	0.5317818
3	*POLD1, CTD-2545M3.6*	POLD1:polymerase (DNA directed), delta 1, catalytic subunit	0.52976793
4	*RECQL4*	RecQ like helicase 4	0.5265696
5	*NRM*	nurim (nuclear envelope membrane protein)	0.50892645
6	*MCM2*	minichromosome maintenance complex component 2	0.50102276
7	*ESPL1*	extra spindle pole bodies like 1, separase	0.49512082
8	*MYBL2*	v-myb avian myeloblastosis viral oncogene homolog-like 2	0.4910358
9	*C19orf48, SNORD88B*	C19orf48:chromosome 19 open reading frame 48, SNORD88B:small nucleolar RNA, C/D box 88B	0.4903525
10	*CDCA8*	cell division cycle associated 8	0.4883732

11	*POLE*	polymerase (DNA directed), epsilon, catalytic subunit	0.48658773
12	*TCF19*	transcription factor 19	0.4831117
13	*CENPM*	centromere protein M	0.46817404
14	*ATAD3B*	ATPase family, AAA domain containing 3B	0.46814716
15	*RNASEH2A*	ribonuclease H2 subunit A	0.4660578
16	*POC1A*	POC1 centriolar protein A	0.46444392
17	*KIF2C*	kinesin family member 2C	0.4628259
18	*RNF26*	ring finger protein 26	0.45479703
19	*RAD54L*	RAD54-like (S. cerevisiae)	0.45234054
20	*KIFC1*	kinesin family member C1	0.45149833
21	*FANCA*	Fanconi anemia complementation group A	0.44833374
22	*RCCD1*	RCC1 domain containing 1	0.44649145
23	*LAGE3P1*	L antigen family member 3 pseudogene 1	0.44513708
24	*MYO19*	myosin XIX	0.4434382
25	*POLA2*	polymerase (DNA directed), alpha 2, accessory subunit	0.44328704

S3 Table. *CCHCR1* co-expressed genes. Data retrieved using Genevestigator [9]. Top 25 positively correlated genes were shown. Repeated gene entry in the data is due to multiple microarray probes of the same gene being used in different studies. NYD: Not yet determined.
