# Supplementary material for: Cell cycle regulation of the psoriasis associated gene CCHCR1 by transcription factor E2F1
Source: PLoS One. 2023 Dec 21;18(12):e0294661. doi: 10.1371/journal.pone.0294661 (PMC10734992; doi:10.1371/journal.pone.0294661)
Supplement: S4 Table — (PDF) [file pone.0294661.s004.pdf]

**Gene**	**Primer sequences (5’ – 3’)**	**Amplicon size (bp)**
RPL13A	AGATGGCGGAGGTGCAG	128
	GTTGATGCCTTCACAGCGTA	
CCHCR1	GGAAGAACTTGGAAGAGGGG	141
	GAGACTTCTCCAAGCCCTCA	
TCF19	GGTGATGACTGGAGGGTCAG	111
	CAGGAGGTCTCCATCACTCA	
E2F1	CCAGGAAAAGGTGTGAAATC	74
	AAGCGCTTGGTGGTCAGATT	

S4 Table. Primers for quantitative PCR.
