# Supplementary figures and images for: Cell cycle regulation of the psoriasis associated gene CCHCR1 by transcription factor E2F1
Source: PLoS One. 2023 Dec 21;18(12):e0294661. doi: 10.1371/journal.pone.0294661 (PMC10734992; doi:10.1371/journal.pone.0294661)

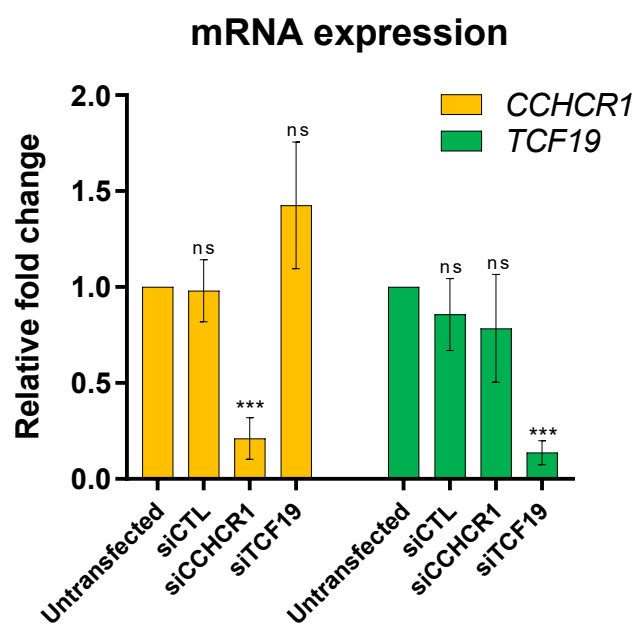

**S1 Fig. siRNAs control experiment.**

Supplement: S1 Fig — (PDF) [file pone.0294661.s005.pdf]
