# Supplementary material for: Cell cycle regulation of the psoriasis associated gene CCHCR1 by transcription factor E2F1
Source: PLoS One. 2023 Dec 21;18(12):e0294661. doi: 10.1371/journal.pone.0294661 (PMC10734992; doi:10.1371/journal.pone.0294661)
Supplement: S1 File — (PDF) [file pone.0294661.s006.pdf]

References

1. Kitao S, Ohsugi I, Ichikawa K, Goto M, Furuichi Y, Shimamoto A. Cloning of two new human helicase genes of the RecQ family: biological significance of multiple species in higher eukaryotes. Genomics. 1998;54(3):443-52. doi: 10.1006/geno.1998.5595. PubMed PMID: 9878247.
2. Corpet A, De Koning L, Toedling J, Savignoni A, Berger F, Lemaitre C, et al. Asf1b, the necessary Asf1 isoform for proliferation, is predictive of outcome in breast cancer. The EMBO journal. 2011;30(3):480-93. doi: 10.1038/emboj.2010.335. PubMed PMID: 21179005; PubMed Central PMCID: PMC3034011.
3. Bryan BA, Dyson OF, Akula SM. Identifying cellular genes crucial for the reactivation of Kaposi's sarcoma-associated herpesvirus latency. The Journal of general virology. 2006;87(Pt 3):519-29. doi: 10.1099/vir.0.81603-0. PubMed PMID: 16476973.
4. Ku DH, Chang CD, Koniecki J, Cannizzaro LA, Boghosian-Sell L, Alder H, et al. A new growth-regulated complementary DNA with the sequence of a putative trans-activating factor. Cell growth & differentiation : the molecular biology journal of the American Association for Cancer Research. 1991;2(4):179-86. PubMed PMID: 1868030.
5. Ivanova AV, Ivanov SV, Lerman ML. Association, mutual stabilization, and transcriptional activity of the STRA13 and MSP58 proteins. Cellular and molecular life sciences : CMLS. 2005;62(4):471-84. doi: 10.1007/s00018-004-4423-2. PubMed PMID: 15719173.
6. Slansky JE, Li Y, Kaelin WG, Farnham PJ. A protein synthesis-dependent increase in E2F1 mRNA correlates with growth regulation of the dihydrofolate reductase promoter. Molecular and cellular biology. 1993;13(3):1610-8. PubMed PMID: 8441401; PubMed Central PMCID: PMC359473.
7. Choudhury AD, Xu H, Baer R. Ubiquitination and proteasomal degradation of the BRCA1 tumor suppressor is regulated during cell cycle progression. The Journal of biological chemistry. 2004;279(32):33909-18. doi: 10.1074/jbc.M403646200. PubMed PMID: 15166217.
8. Fox EJ, Wright SC. The transcriptional repressor gene Mad3 is a novel target for regulation by E2F1. The Biochemical journal. 2003;370(Pt 1):307-13. doi: 10.1042/BJ20021583. PubMed PMID: 12444919; PubMed Central PMCID: PMC1223166.
9. Hruz T, Laule O, Szabo G, Wessendorp F, Bleuler S, Oertle L, et al. Genevestigator v3: a reference expression database for the meta-analysis of transcriptomes. Advances in bioinformatics. 2008;2008:420747. doi: 10.1155/2008/420747. PubMed PMID: 19956698; PubMed Central PMCID: PMC2777001.
