# Supplementary material for: Cell cycle regulation of the psoriasis associated gene CCHCR1 by transcription factor E2F1
Source: PLoS One. 2023 Dec 21;18(12):e0294661. doi: 10.1371/journal.pone.0294661 (PMC10734992; doi:10.1371/journal.pone.0294661)
Supplement: Attachment — Submitted filename: Reviewer for Manuscript Number PONE-D-23-25477.pdf [file pone.0294661.s007.pdf]

In this manuscript the authors investigated the genomic arrangement of CCHCR1 gene. They demonstrate that CCHCR1 shares a bidirectional promoter with its neighboring gene TCF19 and that this bidirectional promoter is activated by the G1/S-regulatory transcription factor E2F1, and both genes are co-induced during the G1/S transition of the cell cycle.

The study is technically well executed and controlled and provides essential insights into the cell cycle regulation of CCHCR1. The data fit well with the topics of PLOS ONE.

Therefore, I think that, once solved some minor issue, this manuscript is suitable for publication on PLOS ONE

Minor point:

-Some concerns with the data showing the expression peak of TCF19 at the G1/S transition. In Figure 2C the increased expression at G1/S transition does not seems clear to me. TCF19 levels at time 0h after double thymidine release are very similar to 20h level. The authors may want to strengthen this claim with additional experiments or add a comment about the expression level of TCF19 at 20h post release.

-In order to make the statement, "Specifically, the long fragment in the forward orientation for CCHCR1 demonstrates higher activity across all cell cycle phases, unlike the corresponding fragment in the reverse orientation for TCF19", a statistic analysis between the luciferase activity of the long promoter fragment in the forward orientation for CCHCR1 and the luciferase activity of the corresponding promoter fragment in the reverse orientation for TCF19 needs to be performed. Only if it is significant that statement might be assessed. Otherwise, it is possible a statement of the observation.

.
